# Factors affecting long‐term survival of non‐small‐cell lung cancer patients with concomitant type 2 diabetes mellitus

**DOI:** 10.1111/1759-7714.14732

**Published:** 2022-11-15

**Authors:** Ju‐Ying Jiang, Chen‐Xiong Hsu, Sang‐Hue Yen

**Affiliations:** ^1^ Division of Endocrinology and Metabolism, Department of Internal Medicine Far Eastern Memorial Hospital New Taipei City Taiwan; ^2^ Division of Radiation Oncology, Department of Radiology Far Eastern Memorial Hospital New Taipei City Taiwan; ^3^ Department of Radiation Oncology Wan Fang Hospital, Taipei Medical University Taipei Taiwan

To the Editor,

We read with interest the study of a retrospective propensity scores matching analysis by Li and colleagues^1^ investigating the effects of type 2 diabetes mellitus (T2DM) on the long‐term survival of patients with stage IIIB‐IV non‐small‐cell lung cancer (NSCLC). The authors report that concomitant T2DM (hazard ratio 1.357, 95% confidence interval 1.121–1.644) and complications by T2DM (hazard ratio 1.623, 95% confidence interval 1.064–2.475) are independent prognostic factors of worse survival in patients with stage IIIB–IV NSCLC. We thoroughly support their conclusion that in addition to dedicated anticancer treatments for stage IIIB‐IV NSCLC patients, further pre‐treatment surveillance and post‐treatment monitoring for those with concomitant T2DM are vitally required. Nevertheless, there are still some issues that need to be addressed.

Glycated hemoglobin (HbA1c) is a reliable biomarker which reflects average endogenous exposure to glucose over approximately 3 months and has a strong predictive value for diabetes‐related microvascular and macrovascular complications.^2^, ^3^, ^4^ In addition, longer diabetes duration means more complications.^5^ Moreover, not only the concomitant T2DM but also the complications severity of diabetes is associated with poor survival in patients with both lung cancer and T2DM.^6^ The adapted diabetes complications severity index (aDCSI) is one of the most useful indicators for assessing diabetes severity. Recently, aDCSI ≥2 has been reported as an independent prognostic factor for the overall survival of patients with both T2DM and stage IIIB–IV NSCLC.^6^

Taken together, the impacts of HbA1c, durations of diabetes, presence of microvascular or macrovascular complications, and severity of diabetes on survival in NSCLC patients with concomitant T2DM remain unclear. Finally, we thank the Li et al. for their excellent work and look forward to more prospective clinical trials to clarify the prognostic factors to improve both diabetes control and survival of NSCLC patients.

## AUTHOR CONTRIBUTION

All authors contributed to the article and approved the submitted version.

## FUNDING INFORMATION

This letter received no financial support.

## CONFLICT OF INTEREST

The authors declare no conflict of interest.
